# The effect of home‐based neuromuscular electrical stimulation‐resistance training and protein supplementation on lean mass in persons with spinal cord injury: A pilot study

**DOI:** 10.14814/phy2.70073

**Published:** 2024-10-02

**Authors:** Sven Hoekstra, James A. King, Jordan Fenton, Natasha Kirk, Scott A. Willis, Stuart M. Phillips, Nick Webborn, Keith Tolfrey, Johan De Vogel‐Van Den Bosch, Vicky L. Goosey‐Tolfrey

**Affiliations:** ^1^ Peter Harrison Centre for Disability Sport, School of Sport, Exercise and Health Sciences Loughborough University Leicestershire UK; ^2^ Department of Exercise and Sport Science St. Mary's University San Antonio Texas USA; ^3^ Department of Rehabilitation Medicine University of Texas Health Science Center at San Antonio San Antonio Texas USA; ^4^ School of Sport, Exercise and Health Sciences Loughborough University Leicestershire UK; ^5^ National Institute for Health and Care Research (NIHR) Leicester Biomedical Research Centre, University Hospitals of Leicester NHS Trust and University of Leicester Leicester UK; ^6^ Department of Kinesiology McMaster University Hamilton Ontario Canada; ^7^ Danone Research and Innovation Utrecht The Netherlands

**Keywords:** exercise training, muscle mass, paralysis, rehabilitation, whey protein

## Abstract

In persons with a spinal cord injury (SCI), resistance training using neuromuscular electrical stimulation (NMES‐RT) increases lean mass in the lower limbs. However, whether protein supplementation in conjunction with NMES‐RT further enhances this training effect is unknown. In this randomized controlled pilot trial, 15 individuals with chronic SCI engaged in 3 times/week NMES‐RT, with (NMES+PRO, *n* = 8) or without protein supplementation (NMES, *n* = 7), for 12 weeks. Before and after the intervention, whole body and regional body composition (DXA) and fasting glucose and insulin concentrations were assessed in plasma. Adherence to the intervention components was ≥96%. Thigh lean mass was increased to a greater extent after NMES+PRO compared to NMES (0.3 (0.2, 0.4) kg; *p* < 0.001). Furthermore, fasting insulin concentration and Homeostatic Model Assessment for Insulin Resistance (HOMA‐IR) were decreased similarly in both groups (fasting insulin: 1 [−9, 11] pmol∙L^−1^; HOMA‐IR: 0.1 [−0.3, 0.5] AU; both *p* ≥ 0.617). Twelve weeks of home‐based NMES‐RT increased thigh lean mass, an effect that was potentiated by protein supplementation. In combination with the excellent adherence and apparent improvement in cardiometabolic health outcomes, these findings support further investigation through a full‐scale randomized controlled trial.

## 1 INTRODUCTION

Spinal cord injury (SCI) results in profound reductions in skeletal muscle mass (SMM) below the lesion level, leading to various metabolic alterations and an increased risk of cardiometabolic diseases (Gorgey & Dudley, 2003). In the initial 6 months post‐injury, lower limb muscle cross‐sectional area is reduced by approximately two‐thirds (Castro et al., 1999). This decline in SMM continues over the first 12 months post‐injury before reaching a plateau (Giangregorio & McCartney, 2006; Gorgey et al., 2014). The loss of SMM results in metabolic changes including increased intramuscular fat, reduced resting metabolic rate (RMR), and decreased muscle oxidative capacity, all contributing to secondary pathologies such as obesity, cardiovascular disease, type 2 diabetes, and dyslipidemia (Biering‐Sorensen et al., 2009; Buchholz & Pencharz, 2004; Cragg et al., 2013; Eisenberg et al., 2022; Gorgey & Dudley, 2003; Koyuncu et al., 2017).

Resistance training using neuromuscular electrical stimulation (NMES‐RT) of the quadriceps is a promising intervention to increase SMM in the paralyzed limbs of people with chronic, motor‐complete SCI (Fenton et al., 2003). A range of studies have reported substantial increases in quadriceps muscle cross‐sectional area with NMES‐RT protocols performed over 8–16 weeks (Gorgey et al., 2012, 2017; Gorgey & Shepherd, 2010; Mahoney et al., 2005; Ryan et al., 2013), oftentimes accompanied by metabolic benefits such as reduced intramuscular fat and improved glucose tolerance (Gorgey et al., 2017; Gorgey & Shepherd, 2010; Mahoney et al., 2005; Ryan et al., 2013). Notwithstanding the robust evidence underpinning the hypertrophic effect of NMES‐RT for persons with SCI, there remains a limited knowledge base for the prescription of *home‐based* protocols. Home‐based NMES‐RT has the potential to be more widely accessible and may therefore help bring down barriers to exercise participation in persons with SCI (Williams et al., 2014). Previous trials investigating home‐based NMES‐RT all induced hypertrophy of the thigh, while metabolic health outcomes were more variable (Gorgey et al., 2017; Mahoney et al., 2005; Ryan et al., 2013). For instance, Gorgey et al. (2017) reported a ~ 15% reduction in intramuscular fat in the thigh, while Ryan et al. (2013) found no improvement in glucose tolerance but an increase in high‐density lipoprotein (HDL)‐cholesterol concentration. These studies investigated a twice per week NMES‐RT protocol, necessitating the exploration of higher‐volume interventions for improvements in cardiometabolic outcomes.

Protein supplementation in conjunction with resistance training augments muscle hypertrophy in healthy adults without a disability (Nunes et al., 2022). Strategies including a combination of resistance training and a high‐protein diet (~1.6 g∙kg^−1^∙day^−1^) have been shown to mitigate atrophy and increase SMM in older populations with sarcopenia (McKendry et al., 2020). However, studies investigating the additive effect of protein supplementation to resistance training in populations experiencing muscle loss due to chronic conditions or disease, such as cancer survivors, individuals with chronic obstructive pulmonary disease (COPD), and those with HIV, show equivocal results (Agin et al., 2001; Constantin et al., 2013; Krull et al., 2020; Madzima et al., 2017). Due to a lack of previous research, the uncertainty around the additive effect of protein supplementation extends to individuals with SCI (Fenton et al., 2003). Yarar‐Fisher et al. (2018) showed that protein supplementation alone is not sufficient to alter SMM in persons with SCI, but similar findings have been observed in healthy adults without a disability after protein supplementation in the absence of RT (Ten Haaf et al., 2018). Given the limited body of literature to date but the paramount importance of identifying effective strategies to maximize SMM to mitigate cardiometabolic disease risk in individuals with SCI, a direct study into the compounding effects of NMES‐RT and protein supplementation is needed.

We investigated the effects of 12 weeks of home‐based NMES‐RT, with (NMES+PRO) and without protein (NMES) supplementation, on lower limb lean mass and cardiometabolic markers in individuals with chronic, motor complete SCI. We hypothesized that NMES+PRO leads to greater gains in thigh lean mass and improvements in glucose tolerance and the lipid profile compared to NMES alone. A secondary objective was to explore the feasibility of performing NMES‐RT in a remotely supervised environment for 12 weeks and the acceptability of consuming a daily protein supplement.

## 2 MATERIALS AND METHODS

### 2.1 Participants

Individuals with motor complete, chronic SCI (male/female, aged 18–60, ≥1‐year post‐injury, American Spinal Injury Association Impairment Scale (AIS) A or B, and lesion level T12 or above) were recruited. Participants were excluded if they had a diagnosed long‐term condition (e.g., diabetes, hypertension), were taking medications known to affect study outcomes, or had contraindications to exercise. Individuals were also excluded if they had performed NMES‐RT once or more per week in the last 6 months, had a habitual protein intake exceeding 2 g∙kg^−1^∙day^−1^.

Prior to enrolment, participants were virtually screened for eligibility via video conferencing software (Microsoft Teams, USA) and were asked to complete a Physical Activity Readiness Questionnaire (2021 PAR‐Q+) (Warburton et al., 2021) and an Institutional Health Screen Questionnaire. These forms were reviewed by a medical professional (NW) and an IRMER certified referrer who authorized participants' involvement in the NMES‐RT and dual energy x‐ray absorptiometry (DXA) scan. We did not assess participants for lower motor neuron damage. This study was approved by the Loughborough University Ethics Review Sub‐Committee (Project ID: 3176) and was registered a priori on Clinical Trials (NCT05249985). All participants gave written informed consent before participation.

### 2.2 Experimental design and randomization

This study was designed as a randomized active‐controlled study, with the following two study arms: NMES with protein supplementation (NMES+PRO) and NMES only (NMES). Participants were randomized to a group using an online randomization tool (https://www.random.org/). As muscular adaptations to NMES‐RT might be different between males and females, participants were stratified by sex to achieve similar baseline conditions (Roberts et al., 2020). Due to the nature of the study, blinding was not possible.

## 3 INTERVENTIONS

### 3.1 Neuromuscular electrical stimulation resistance training (NMES‐RT)

Exercise was performed thrice weekly for 12 weeks with at least 24 h recovery using an NMES device (Fit 3.0, Compex, Écublens, Switzerland). Participants conducted sessions remotely at home with virtual monitoring from one of the research team members (JF, NK). The stimulation targeted the left and right knee extensor (quadriceps) muscles simultaneously while the participant was seated. Electrodes were placed distally on the vastus medialis, midway along the vastus lateralis (5 × 5 cm), and proximally across the vastus intermedius (5 × 10 cm) to allow for knee extension and flexion. Subsequently, electrical square‐wave stimuli within the 35 Hz and 300 μs pulse width were delivered to the participants' left and right thighs using the “Muscle Atrophy” program of the NMES device. The amplitude for both legs was gradually increased until knee extension was achieved. The minimum amplitude needed to reach knee extension throughout the training program was 50 mA, and the maximum was 999 mA. Participants positioned their knees at a 90° angle (starting angle) with their feet slightly elevated from the floor. They gradually increased the amplitude until both legs were fully extended (0 flexion in the knee), or until the device had reached maximum capacity, unless prohibited by leg spasm. Each repetition lasted 5 s and was separated by a 5 s rest phase. Once 10 repetitions had been completed, the program was paused for 3–4 min recovery phase before starting the next set. Four sets of 10 repetitions were completed in each session. All participants began the training intervention with no resistance and 0.5 kg ankle weights (DKN, London, England) were added each time 40 repetitions were completed to full extension. If 40 full repetitions were not achieved, participants maintained their current load for the subsequent session. The amplitude of the stimulation device on each set was recorded to ensure that an increase in external load was due to muscle adaptations rather than an increase in intensity.

### 3.2 Protein supplementation

Participants in the NMES+PRO group were asked to consume a daily leucine‐enriched whey protein supplement (628 kJ, consisting of 21 g protein, 3 g total leucine, 9 g carbohydrate, 3 g fat; Danone, Zoetermeer, the Netherlands) in addition to undertaking the NMES‐RT intervention. Participants were advised to consume the protein supplement after each training session but were free to choose the timing of the supplement consumption on the remaining days; however, the timing of the protein consumption was not specifically monitored. Adherence to the protein supplementation protocol was self‐reported and verbally checked at each session by the research team. Throughout the intervention, participants were reminded not to alter their diet or physical activity behavior. Full protein supplement details are found in the Table S1.

In the week before both laboratory visits, participants completed a three‐day weighed food diary, a seven‐day sleep diary, and wore an accelerometer watch on their non‐dominant wrist. Each session consisted of obtaining RMR in a supine position while blood pressure was measured. Participants were then moved to a reclined position and a 21G cannula (Venflon, Becton Dickinson, Helsingborg, Sweden) was inserted into an antecubital vein. A fasted blood sample was obtained to determine circulating concentrations of glucose, insulin, triglycerides, total cholesterol, low‐density lipoprotein‐cholesterol, and HDL‐cholesterol. Participants subsequently underwent an oral glucose tolerance test (OGTT), in which they consumed a 75‐g glucose drink (300 g water, 82.5 g maltodextrin [MyProtein, Manchester, UK]) with subsequent blood samples collected at 15, 30, 45, 60, 90, and 120 min thereafter for the determination of circulating insulin and glucose concentrations. Whole body composition was assessed using DXA. Finally, in the baseline trial, a 30 min practice of RT using NMES was conducted to familiarize participants with the stimulation device.

## 4 OUTCOMES

**FIGURE 1:**
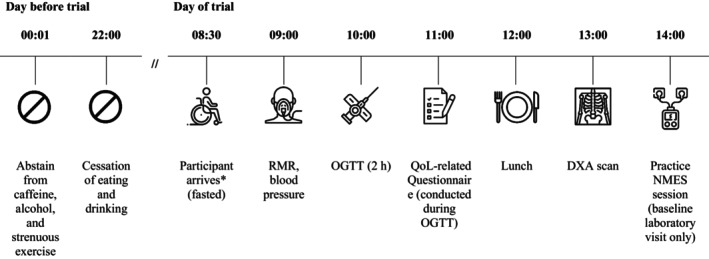
Schematic illustration of the baseline and follow‐up laboratory experiment protocol (DXA, dual‐energy x‐ray absorptiometry; NMES, neuromuscular electrical stimulation; OGTT, oral glucose tolerance test; QoL, quality of life; RMR, resting metabolic rate). *Between 08:30–09:00, participants sign study forms, go to the toilet, are re‐familiarized with the procedures, and transfer to the bed.

Outcomes were assessed in the laboratory at baseline and within 7 days after the two 12‐week NMES‐RT interventions. For each visit, participants arrived at the laboratory at 08:30 (Figure 1); participants who lived >1 h drive from the laboratory slept in a hotel adjacent to the university. The day before each visit, participants were instructed to abstain from caffeine, alcohol and strenuous exercise, and cease eating by 22:00. During the laboratory visits, a pasta lunch consisting of pasta, tomato sauce, and shredded cheddar (44% carbohydrate, 41% fat, 14% protein) was provided. The quantity consumed was measured and standardized between both visits.

### 4.1 Resting metabolic rate and blood pressure

RMR was determined using indirect calorimetry using a mobile spiroergometric device (Metamax 3B, Cortex, Leipzig, Germany) in accordance with best practice guidelines (Compher et al., 2006). Briefly, the device was calibrated in line with the manufacturer's instructions against atmospheric pressure, a standardized gas concentration (15% O_2_ and 5% CO_2_) and participant anthropometry via the MetaSoft Studio software (Cortex, Leipzig, Germany). A face mask (Hans Rudolph incorporated, Kansas, USA) was fitted to the participant, including a disposable turbine volume transducer (Cortex, Leipzig, Germany) and sample line (Perma Pure LLC, New Jersey, USA) connected into the Metamax 3B system. Participants lay in a supine position for 30 min while the device measured oxygen consumption (V̇O_2_), carbon dioxide output (V̇CO_2_), and the volume of expired air on a second‐by‐second basis. The final 10 min were used for analysis. Carbohydrate and fat oxidation were determined from the ratio of V̇O_2_ and V̇CO_2_, with the assumption that protein oxidation at rest was negligible (Frayn, 1983). Energy expenditure was then calculated using a caloric equivalent for carbohydrate and fat oxidation and averaged over the last 10 min. This was extrapolated over 24 h to determine RMR. Participants remained in a supine position while blood pressure was assessed via an automated blood pressure device (Omron, Kyoto, Japan) on the minute, every minute, for 4 min and an average of the four values recorded.

### 4.2 Anthropometric measures

In a fasted state and after participants had voided their bladder, body mass was measured in minimal clothing to the nearest 0.1 kg using a wheelchair weighing scale (DETECTO 6550, Missouri, USA). Height was measured in the supine position to the nearest 0.1 cm with a steel Lufkin measuring tape. Waist and hip circumference were assessed in duplicate and in accordance with previous studies in SCI populations (Gill et al., 2020) using an inelastic polyfibre tape measure (Seca, Birmingham, England).

### 4.3 Dual x‐ray absorptiometry

Whole body composition was assessed on a GE Lunar iDXA (GE Healthcare, Illinois, USA) (software: enCORE Version 16) to measure lean mass, fat mass, and total‐body bone mineral density (BMD). The scanner was calibrated daily using standard protocols, and an aluminum spine phantom also scanned daily to ensure there was no drift. Protocols for whole body scan acquisition in persons with SCI have been described previously (Keil et al., 2016). All participants wore light clothing containing no metal artifacts, and participants with a catheter bag detached the bag from their leg prior to the scan. Regions of interest were automatically determined for whole body, legs, and trunk, and were checked manually and adjusted to remove any non‐tissue artifacts. To determine the composition of the thighs, a custom analysis was drawn manually by two trained personnel around both legs from the bottom edge of the ischium bone, traversing through the femoral neck, to the distal edge of the tibiofemoral joint. All custom analyses were performed in duplicate (all mean CVs ≤1.23%). Average compositions of the right and left legs and thighs were recorded. For participants with metal implants in their legs, values from the leg without implants were recorded. Due to a change in study personnel, the final four participants (two in each group) were analyzed by OC, while the others were analyzed by JF.

### 4.4 Biochemical analyses

Blood samples were drawn into ice‐cooled K_3_EDTA monovettes (Sarstedt, Nümbrecht, Germany) and immediately centrifuged (10 min, 2383 *g*, 4°C). Plasma was extracted into 2 mL cryovial tubes and stored at −80°C for future analysis. The cannula was kept patent by flushing with saline solution (10 mL of 0.9% sodium chloride w/v) every 15 min. Commercially available enzyme‐linked immunosorbent assays were used to determine plasma concentrations of insulin (Mercodia AB, Uppsala, Sweden, 10–1113‐01), and enzymatic, colorimetric methods (using a bench‐top analyzer) were used to determine circulating concentration of glucose (A11A01668), triglycerides (A11A01640), cholesterol (A11A01634), low‐density lipoprotein (LDL)‐cholesterol (A11A01638), and HDL‐cholesterol (A11A01636) (Pentra 400; Horiba Medical, Northampton, England; all mean within‐batch CVs ≤1.98%). The mean, intra‐plate CV for insulin was 4.4%. Insulin resistance and sensitivity were assessed by HOMA‐IR (Matthews et al., 1985) and the Matsuda Insulin Sensitivity Index (ISI) (Matsuda & DeFronzo, 1999).

### 4.5 Sleep, physical activity, and quality of life

Seven days before each laboratory visit, participants wore a tri‐axial accelerometer (GENEActiv, Activinsights, Huntingdon, England) on their non‐dominant wrist to record habitual light, moderate, and heavy physical activity levels, sedentary time, and sleep quantity and efficiency. This device has been validated in manual wheelchair users (Nightingale et al., 2015). To ensure that predicted sleep onset and duration were correct according to the GENEActiv device, participants completed a corresponding sleep log detailing the time participants went to bed, tried to go to sleep, woke up, and the time they got out of bed.

Participants were asked to complete questionnaires related to leisure time physical activity (Ginis et al., 2012), neuropathic pain (Krause & Backonja, 2003), subjective health‐related quality of life (Hays et al., 1993), sleep quality, (Yi et al., 2006) and spasticity (Adams et al., 2007). In the follow‐up trial, participants also completed a bespoke end‐of‐intervention Likert‐scale questionnaire to answer generic questions about their personal experience with using NMES and experiences of ingesting protein supplements. Answers ranged from “strongly disagree” to “strongly agree.” This was followed by open‐ended questions which allowed participants to elaborate on any of the previous multiple‐choice questions and provide feedback on the intervention.

## 5 STATISTICAL ANALYSIS

Normality of the data was assessed using visual inspection of histograms and the Shapiro–Wilk test. Baseline characteristics of the intervention groups are reported as mean ± SD for normally distributed continuous data, median (interquartile range) for non‐normally distributed continuous data, and number [percentage] for categorical data. Total incremental area under the curve (AUC) values for glucose and insulin concentrations during the two‐hour OGTT were calculated using the trapezoidal method. The primary outcome was the difference in the change (post‐ minus pre‐intervention values) in thigh lean mass between the interventions, assessed using generalized linear models (GLMs) with a normal distribution and identity link function, with the intervention group (binary indicator) as the explanatory variable and the baseline (pre‐intervention) value of the outcome as a covariate. A complete‐case approach was utilized, while a sensitivity analysis of the primary outcome was also conducted using an intention‐to‐treat approach with multiple imputation for missing values. Missing data were imputed across 20 datasets using a regression model with the following predictors: age, AIS grade, neurological level of injury, BMI, intervention group, and baseline value of the outcome (Bourguignon et al., 2024). All secondary outcomes were analyzed using GLMs and the primary analysis outlined above. Model results are presented as adjusted means with 95% confidence intervals (CIs) for within‐group changes and the between‐group difference (NMES+PRO minus NMES). *p*‐values and effect sizes (ES; Cohen's *d*) are presented for between‐group differences, with thresholds of *d* = 0.2, 0.5, and 0.8 denoting a small, moderate and large ES, respectively (Cohen, 1998). Within‐group analyses are inferred from 95% CI. Differences in the ankle weights used at the end of the 12 weeks between groups were assessed using an independent *t*‐test. All analyses were performed using SPSS Statistics v29 (SPSSS Inc., Illinois, USA) and statistical significance was set at an alpha level of *p* < 0.05.

## 6 RESULTS

### 6.1 Participants and missing data

**FIGURE 2:**
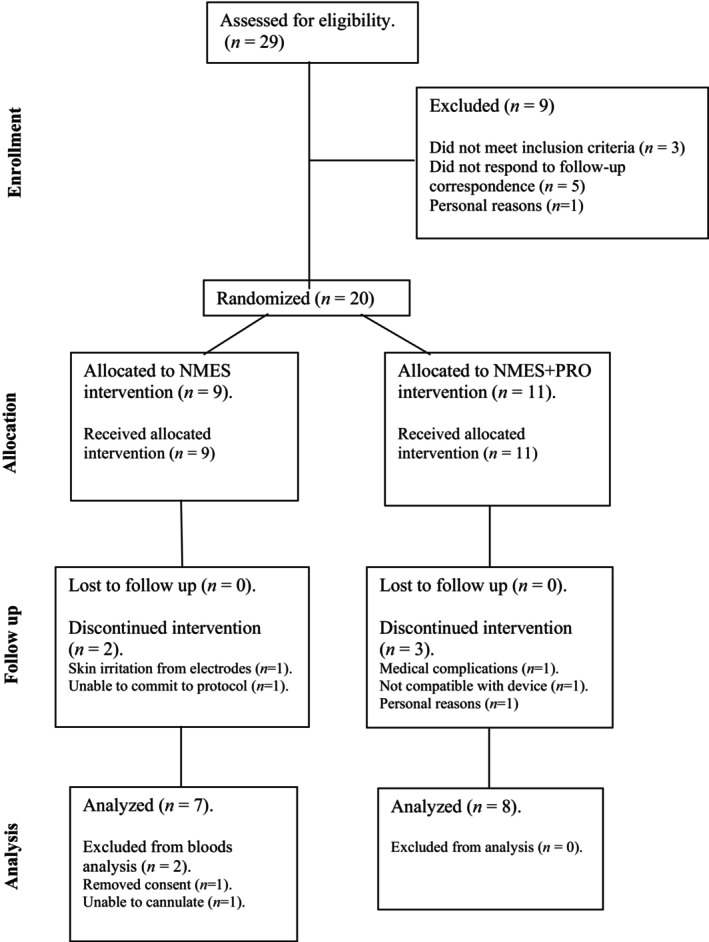
CONSORT diagram illustrating the flow of participants through the neuromuscular electrical stimulation (NMES) and neuromuscular electrical stimulation + protein supplementation (NMES+PRO) study arms.

The CONSORT flow diagram is presented in Figure 2. Between April 2022 and February 2024, a total of 29 individuals registered their interest in the study. After screening, 20 participants enrolled in the study and were randomly allocated to either the NMES (*n* = 9) or NMES+PRO (*n* = 11) interventions. Across the 12 weeks, two participants undertaking NMES and three participants undertaking NMES+PRO discontinued the interventions; thus, 15 participants completed the study (*n* = 7 for NMES and *n* = 8 for NMES+PRO). Due to technical issues with the intravenous cannulation procedure, blood data are available for 13 participants (*n* = 5 for NMES and *n* = 8 for NMES+PRO).

Baseline characteristics of participants in the NMES and NMES+PRO groups are shown in Table 1. Collectively, participants were predominantly male (93%), had an AIS grade of either A (73%) or B (27%), and had a neurological level of injury at either the cervical (60%) or thoracic level (40%). Age and length of time since injury were similar between the two intervention groups; however, some anthropometric and body composition measures (namely body weight, BMI, waist circumference, total body and trunk fat mass, total body and leg lean mass, and total‐body BMD) were higher in the NMES+PRO versus NMES group. Furthermore, participants in the NMES+PRO group had lower HDL‐cholesterol and higher blood pressure, resting heart rate and triglycerides compared to participants in the NMES group. All other anthropometric, body composition, cardiometabolic, and quality of life‐related characteristics were similar between the two intervention groups. Participants' baseline physical activity, sedentary time, sleep and dietary habits are shown in Table S1 and were not different between the two intervention groups.

**TABLE 1:** Baseline (pre‐intervention) participant characteristics of the NMES and NMES+PRO groups.

Variable	NMES (*n* = 7)	NMES+PRO (*n* = 8)
Demographics		
Age (years)	47 ± 11	44 ± 16
Sex		
Female—*n* [%]	1 [14.3]	0 [0]
Male—*n* [%]	6 [85.7]	8 [100]
AIS grade		
A—*n* [%]	6 [85.7]	5 [62.5]
B—*n* [%]	1 [14.3]	3 [37.5]
Neurological level of injury		
Cervical—*n* [%]	5 [71.4]	4 [50.0]
Thoracic—*n* [%]	2 [28.6]	4 [50.0]
TSI (years)	6 (5, 25)	9 (2, 22)
Anthropometry		
Body weight (kg)	73.8 ± 17.7	88.8 ± 8.8
BMI (kg∙m^−2^)	23.0 ± 4.5	26.9 ± 2.2
Waist circumference (cm)	92.6 ± 11.9	101.8 ± 8.2
Hip circumference (cm)	100.2 ± 7.7	104.6 ± 4.6
Waist‐to‐hip ratio (AU)	0.92 ± 0.09	0.97 ± 0.05
Body composition		
Lean mass		
Thigh (kg)	4.4 ± 1.4	5.2 ± 1.2
Leg (kg)	12.8 ± 3.4	16.3 ± 3.2
Trunk (kg)	23.1 ± 4.1	25.8 ± 2.4
Total body (kg)	46.5 ± 9.6	53.8 ± 6.1
Fat mass		
Thigh (kg)	2.8 ± 1.0	3.1 ± 0.7
Leg (kg)	7.4 ± 2.4	8.5 ± 1.8
Trunk (kg)	13.3 ± 6.0	18.2 ± 3.1
Total body (kg)	24.5 ± 9.1	30.8 ± 4.5
BMD		
Total body (g∙cm^−2^)	1.18 ± 0.19	1.33 ± 0.15
Resting metabolic rate, blood pressure, and cardiometabolic outcomes		
RMR (kcal∙day^−1^)	1538 ± 308	1792 ± 372
Blood pressure		
SBP (mmHg)	111 (106, 141)	127 (122, 159)
DBP (mmHg)	69 (59, 84)	85 (64, 95)
Resting heart rate (bpm)	50 ± 7	62 ± 9
Fasting glucose (mmol∙L^−1^)^a^	4.3 ± 0.7	4.8 ± 0.4
Fasting insulin (pmol∙L^−1^)^a^	24 (19, 32)	32 (26, 54)
HOMA‐IR (AU)^a^	1.0 ± 0.4	1.6 ± 0.7
Glucose AUC (mmol∙L^−1^∙2 h)^a^	717 ± 233	867 ± 229
Insulin AUC (nmol∙L^−1^∙2 h)^a^	31 ± 13	41 ± 15
Matsuda ISI (AU)^a^	26.3 ± 6.1	17.6 ± 9.5
Triglycerides (mmol∙L^−1^)^a^	0.79 ± 0.14	1.51 ± 0.31
Total cholesterol (mmol∙L^−1^)^a^	4.39 ± 0.80	4.98 ± 0.77
HDL‐c (mmol∙L^−1^)^a^	1.26 ± 0.17	1.09 ± 0.14
LDL‐c (mmol∙L^−1^)^a^	2.47 ± 0.85	2.93 ± 0.82
Quality of life‐related outcomes		
Physical health limitations	50 (0, 100)	100 (25, 100)
Emotional health limitations	100 (33, 100)	100 (100, 100)
Energy/fatigue	59 ± 21	65 ± 13
Emotional wellbeing	84 (36, 84)	82 (77, 87)
Social functioning	75 (50, 100)	88 (75, 100)
Pain	64 ± 20	59 ± 10
General health	47 ± 25	63 ± 17
Self‐reported sleep quality	31 ± 13	33 ± 7
Spasm severity	−0.51 ± 0.83	−0.35 ± 0.44
Neuropathic pain	−0.28 ± 0.90	−0.06 ± 1.29

*Note*: Categorical data are presented as number [percentage] and continuous data are presented as mean SD, or as median (interquartile range) where data were non‐normally distributed.

Abbreviations: AIS, American Spinal Injury Association Impairment Scale; AUC, area under the curve; BMD, bone mineral density; BMI, body mass index; DBP, diastolic blood pressure; HDL‐c, high‐density lipoprotein cholesterol; HOMA‐IR, homeostatic model assessment of insulin resistance; ISI, insulin sensitivity index; LDL‐c, low‐density lipoprotein cholesterol; NMES, neuromuscular electrical stimulation; NMES+PRO, neuromuscular electrical stimulation + protein supplementation; RMR, resting metabolic rate; SBP, systolic blood pressure; TSI, time since injury.

^a^ *n* = 5 for the NMES group.

### 6.2 Intervention adherence

Adherence to the NMES component of the interventions was 96% (35 ± 2 out of 36 sessions) and 99% (35 ± 1 out of 36 sessions) for NMES and NMES+PRO, respectively. Furthermore, self‐reported adherence to the daily protein supplement in the NMES+PRO group was 97.8%. Ankle weights lifted by the end of the 12‐week interventions were 2.6 ± 2.9 kg in the NMES group and 4.9 ± 4.3 kg in the NMES+PRO group (*p* = 0.252; *d* = 0.621). Four participants (*n* = 2 for both NMES and NMES+PRO) did not use additional ankle weights throughout the intervention as full extension of the legs was never reached.

**FIGURE 3:**
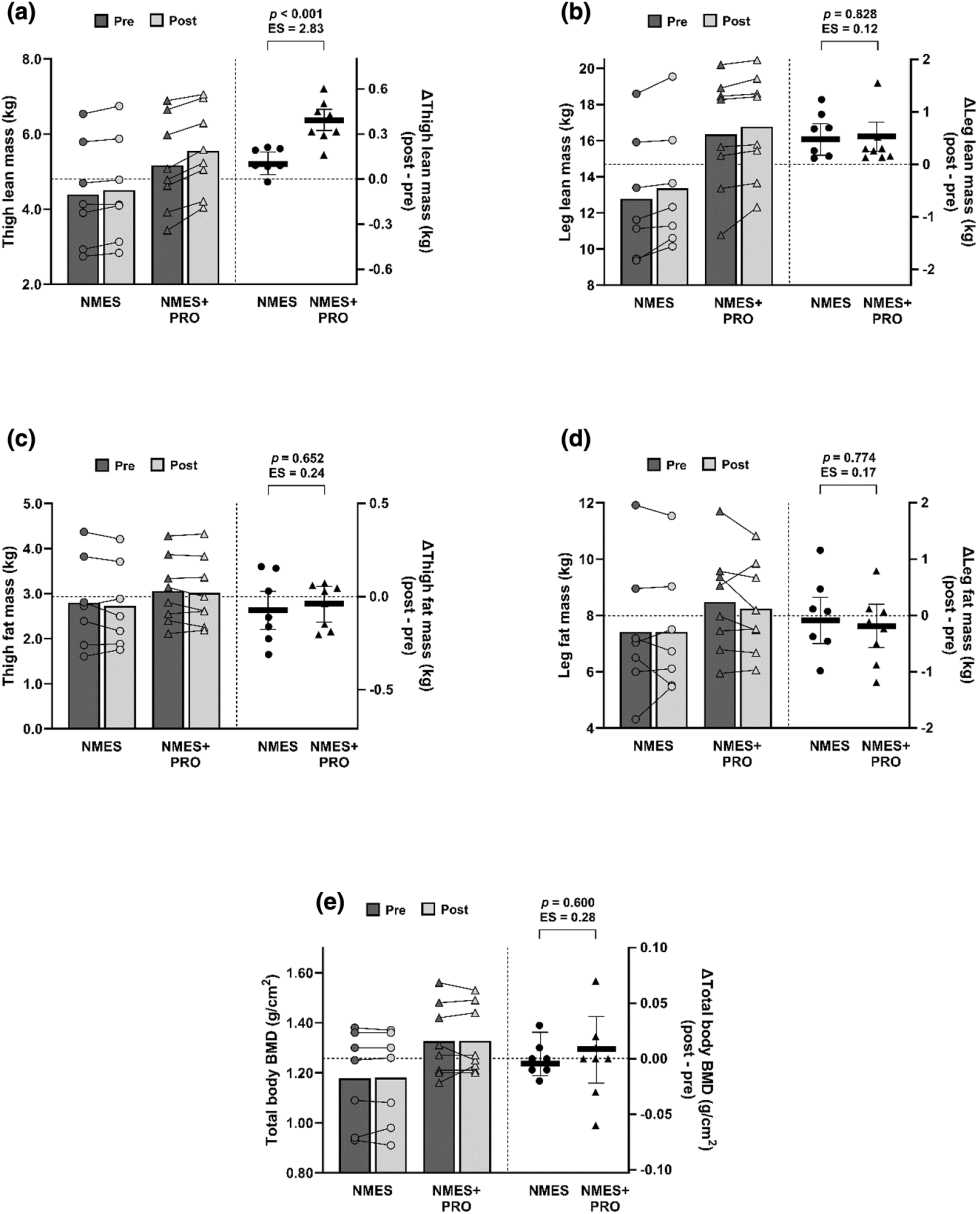
Changes in thigh and leg lean mass (a, b), fat mass (c, d), and total body bone mineral density (BMD; e) in response to the 12‐week neuromuscular electrical stimulation (NMES; circles; *n* = 7) and neuromuscular electrical stimulation + protein supplementation (NMES+PRO; triangles; *n* = 8) interventions. The left‐hand *y* axis displays the raw pre‐ and post‐intervention values for each participant, with the bars representing the corresponding group mean. The right‐hand *y* axis displays the change (post‐ minus pre‐intervention) values for each participant, with the horizontal lines representing the mean change and 95% confidence intervals (CIs) for each intervention group adjusted for baseline (pre‐intervention) values. The *p*‐values and effect sizes (Cohen's *d*) are presented for the between‐group differences (NMES+PRO minus NMES).

**FIGURE 4:**
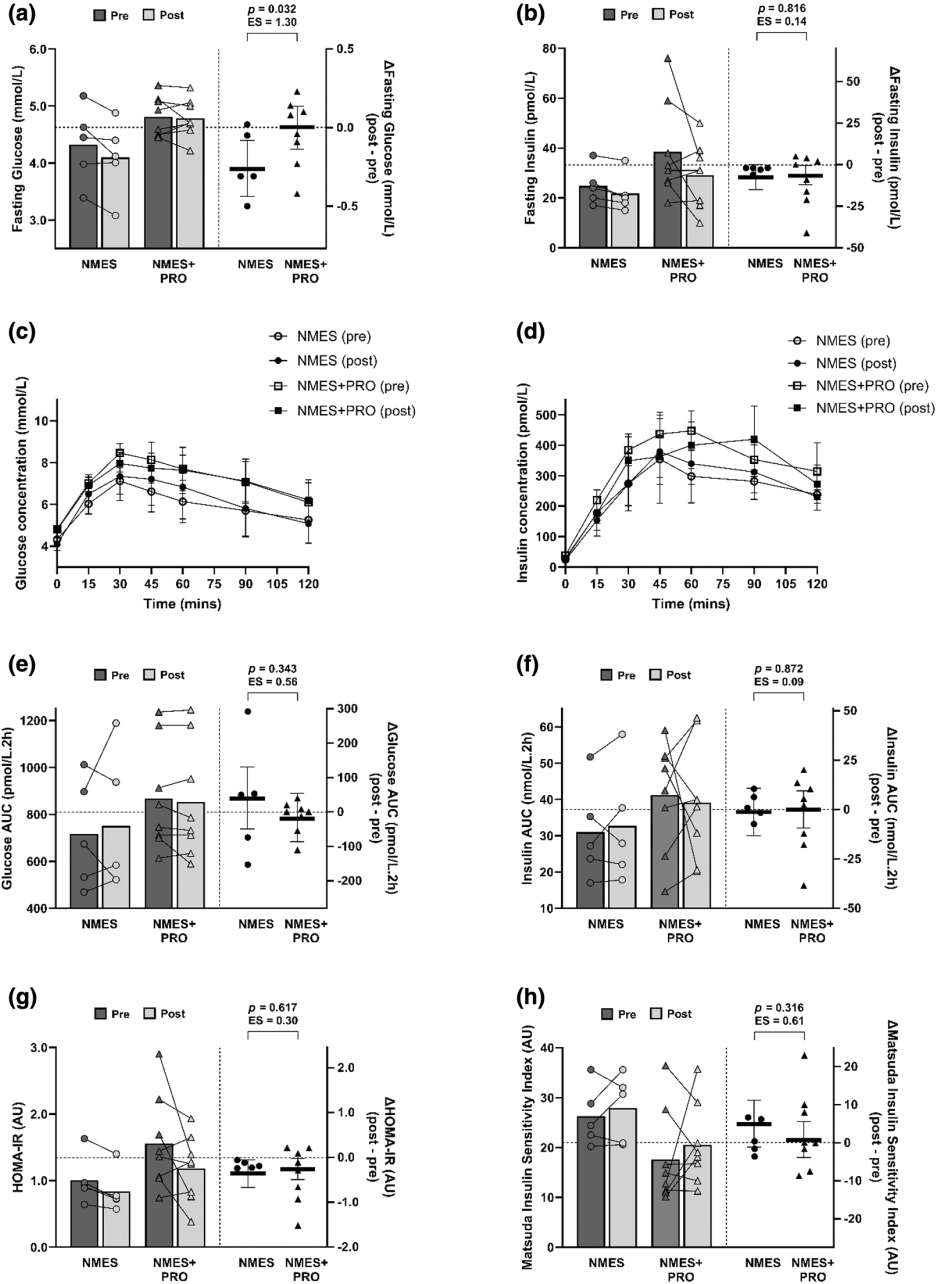
Changes in fasting glucose and insulin concentration (a, b), postprandial glucose and insulin concentrations (c, d), OGTT‐derived glucose and insulin area under the curve (AUC; e, f), homeostatic model assessment of insulin resistance (HOMA‐IR; g) and Matsuda Insulin Sensitivity Index (ISI; h) in response to the 12‐week neuromuscular electrical stimulation (NMES; circles; *n* = 5) and neuromuscular electrical stimulation + protein supplementation (NMES+PRO; triangles; *n* = 8) interventions. The left‐hand *y* axis displays the raw pre‐ and post‐intervention values for each participant, with the bars representing the corresponding group mean. The right‐hand *y* axis displays the change (post‐ minus pre‐intervention) values for each participant, with the horizontal lines representing the mean change and 95% confidence intervals (CIs) for each intervention group adjusted for baseline (pre‐intervention) values. The *p*‐values and effect sizes (Cohen's *d*) are presented for the between‐group differences (NMES+PRO minus NMES).

Changes in anthropometry, body composition, blood pressure, RMR, cardiometabolic outcomes and quality of life‐related outcomes after the 12‐week interventions, and the between‐group differences, are presented in Table 2 and Figures 3 and 4.

**TABLE 2:** Pre‐ to post‐intervention (within‐group) changes and the between‐group differences (NMES+PRO minus NMES) after the 12‐week intervention period.

Variable	Mean change from baseline (post minus pre)				Between‐group difference (NMES + PRO minus NMES) (95% CI)	*p*‐value	Effect size (*d*)
	NMES		NMES + PRO				
	*n*	Mean change (95% CI)	*n*	Mean change (95% CI)			
Anthropometry							
Body weight (kg)	7	0.3 (−1.9, 2.5)	8	−1.1 (−3.2, 0.9)	−1.4 (−4.7, 1.8)	0.384	0.48
BMI (kg∙m^−2^)	7	0.1 (−0.6, 0.8)	8	−0.4 (−1.0, 0.3)	−0.5 (−1.4, 0.5)	0.367	0.50
Waist circumference (cm)	7	−1.1 (−2.8, 0.7)	8	−3.0 (−4.7, −1.4)	−1.9 (−4.5, 0.6)	0.135	0.82
Hip circumference (cm)	7	0.2 (−2.9, 3.4)	8	−0.1 (−3.0, 2.9)	−0.3 (−4.8, 4.2)	0.889	0.07
Waist‐to‐hip ratio	7	−0.02 (−0.05, 0.01)	8	−0.02 (−0.05, 0.01)	−0.00 (−0.04, 0.03)	0.809	0.13
Body composition							
Lean mass							
Trunk (kg)	7	−0.3 (−0.8, 0.2)	8	−0.4 (−0.8, 0.1)	−0.1 (−0.7, 0.6)	0.863	0.09
Total body (kg)	7	0.4 (−0.3, 1.1)	8	0.1 (−0.5, 0.8)	−0.3 (−1.3, 0.8)	0.614	0.27
Fat mass							
Trunk (kg)	7	0.4 (−0.5, 1.3)	8	−0.2 (−1.0, 0.6)	−0.6 (−1.9, 0.7)	0.343	0.52
Total body (kg)	7	0.4 (−0.7, 1.5)	8	−0.5 (−1.6, 0.5)	−0.9 (−2.6, 0.7)	0.271	0.60
BMD							
Total body (g∙cm^−2^)	7	0.00 (−0.02, 0.02)	8	0.00 (−0.02, 0.02)	0.00 (−0.02, 0.04)	0.600	0.28
RMR, blood pressure and cardiometabolic outcomes							
RMR (kcal∙day^−1^)	7	115 (−99, 330)	8	35 (−165, 235)	−80 (−384, 224)	0.605	0.28
Blood pressure							
SBP (mmHg)	7	0 (−8, 7)	8	−4 (−11, 3)	−4 (−14, 7)	0.508	0.35
DBP (mmHg)	7	−1 (−7, 5)	8	0 (−5, 6)	1 (−7, 10)	0.768	0.16
HR (bpm)	7	−4 (−10, 3)	8	−1 (−7, 5)	3 (−7, 12)	0.578	0.32
Triglycerides (mmol∙L^−1^)	5	0.03 (−0.72, 0.78)	8	0.38 (−0.15, 0.90)	0.35 (−0.77, 1.45)	0.543	0.43
Total cholesterol (mmol∙L^−1^)	5	−0.22 (−0.55, 0.11)	8	0.03 (−0.23, 0.29)	0.25 (−0.19, 0.69)	0.262	0.66
HDL‐c (mmol∙L^−1^)	5	0.00 (−0.13, 0.12)	8	−0.06 (−0.16, 0.03)	−0.06 (−0.23, 0.11)	0.462	0.45
LDL‐c (mmol∙L^−1^)	5	−0.30 (−0.55, −0.04)	8	0.05 (−0.15, 0.25)	0.35 (0.02, 0.68)	0.037*	1.21
Quality of life‐related outcomes							
Physical health limitations	7	5 (−20, 30)	8	21 (−2, 44)	16 (−19, 52)	0.363	0.49
Emotional health limitations	7	−17 (−40, 7)	8	10 (−12, 32)	27 (−7, 61)	0.125	0.84
Energy/fatigue	7	0 (−9, 9)	8	1 (−7, 10)	1 (−11, 14)	0.851	0.10
Emotional wellbeing	7	2 (−4, 9)	8	0 (−7, 6)	−2 (−12, 7)	0.596	0.28
Social functioning	7	4 (−9, 17)	8	17 (5, 29)	13 (−5, 30)	0.168	0.72
Pain	7	9 (−10, 27)	8	1 (−16, 18)	−8 (−32, 18)	0.561	0.30
General health	7	7 (−1, 14)	8	3 (−4, 10)	−4 (−14, 6)	0.483	0.38
Self‐reported sleep quality	7	−4 (−9, 1)	8	−5 (−10, 0)	−1 (−7, 6)	0.791	0.14
Spasm severity	7	−0.01 (−0.31, 0.30)	8	0.16 (−0.12, 0.44)	0.17 (−0.25, 0.58)	0.433	0.41
Neuropathic pain	7	−0.02 (−0.33, 0.28)	8	0.17 (−0.12, 0.45)	0.19 (−0.23, 0.61)	0.375	0.46

*Note*: Data were analyzed using generalized linear models with a normal distribution and identity link function and are presented as mean change from baseline adjusted for pre‐intervention values with 95% CI.

Abbreviations: BMD, bone mineral density; BMI, body mass index; CI, confidence interval; DBP, diastolic blood pressure; HDL‐c, high‐density lipoprotein cholesterol; LDL‐c, low‐density lipoprotein cholesterol; NMES, neuromuscular electrical stimulation; NMES+PRO, neuromuscular electrical stimulation + protein supplementation; RMR, resting metabolic rate; SBP, systolic blood pressure.

* *p* < 0.05.

### 6.3 Changes in anthropometry

Body weight, BMI, hip circumference, and waist‐to‐hip ratio were unchanged after both NMES and NMES+PRO (Table 2). The between‐group difference for waist circumference did not reach significance (−1.9 [−4.5, 0.6] cm; *p* = 0.135; *d* = 0.82), despite a within‐group change of −3.0 (−4.7, −1.4) cm after NMES+PRO compared to −1.1 (−2.8, 0.7) cm after NMES (Table 2).

#### 6.3.1 Changes in body composition

After 12 weeks, thigh lean mass was increased to a greater extent after NMES+PRO compared to NMES (mean between‐group difference: 0.3 (0.2, 0.4) kg; *p* < 0.001; *d* = 2.83; Figure 3a). The intention‐to‐treat sensitivity analysis for the primary outcome (thigh lean mass; *n* = 9 for NMES and *n* = 11 for NMES+PRO) revealed the same pattern of results and interpretation (NMES: 0.1 [0.1, 0.2] kg; NMES+PRO: 0.4 [0.4, 0.5] kg; mean between‐group difference: 0.3 [0.2, 0.4] kg; *p* < 0.001; *d* = 3.09). Both interventions also increased leg lean mass by 0.5 (0.2, 0.8) kg; however, no between‐group difference was evident (0.0 [−0.4, 0.5] kg; *p* = 0.828; *d* = 0.12; Figure 3b). Trunk and total body lean mass were unchanged after the NMES and NMES+PRO interventions (Table 2). Similarly, there were no changes in thigh, leg, trunk and total body fat mass in response to the two interventions (Figure 3c,d; Table 2). No changes in total body BMD were observed after the two interventions (Figure 3e; Table 2).

### 6.4 Changes in blood pressure, RMR, and cardiometabolic outcomes

There were no changes in blood pressure, RMR and resting heart rate in response to the NMES, and NMES+PROinterventions (Table 2). Between‐group differences were identified for fasting glucose (0.3[0.0, 0.5] mmol∙L^−1^; *p* = 0.032; *d* = 1.30; Figure 4a) and LDL‐cholesterol (0.35 [0.02, 0.68] mmol∙L^−1^; *p* = 0.037; *d* = 1.21; Table 2) whereby fasting glucose and LDL‐cholesterol concentrations were reduced after NMES and unchanged after NMES+PRO. Furthermore, fasting insulin concentration and HOMA‐IR were decreased similarly after NMES and NMES+PRO, with no between‐group differences (fasting insulin: 1 [−9, 11] pmol∙L^−1^; HOMA‐IR: 0.1 [−0.3, 0.5] AU; both *p* ≥ 0.617; *d* ≤ 0.30; Figure 4b,g). There were no changes in other cardiometabolic outcomes in response to the two interventions (Figure 4c–f,h; Table 2).

### 6.5 Changes in quality of life‐related outcomes

There were no between‐group differences for any questionnaire outcomes relating to quality of life, self‐reported sleep quality, spasm severity, or neuropathic pain (Table 2). However, there was a within‐group change in social functioning score of 17 (Buchholz & Pencharz, 2004; Gill et al., 2020) after NMES+PRO compared to 4 (−9, 17) after NMES (Table 2). Furthermore, self‐reported sleep quality was improved by both the NMES (−4 [−9, 1]) and NMES+PRO (−5 [−9, 0]) interventions (Table 2). No within‐group changes were observed for any other questionnaire outcomes (Table 2).

### 6.6 Changes in physical activity, sedentary time, sleep, and dietary intake

Changes in physical activity, sedentary time, sleep and dietary intake after the 12‐week interventions, and the between‐group differences, are presented in Table S2. No between‐group differences were identified for any outcomes; however, within‐group analyses revealed that energy intake, protein intake and fat intake were decreased after both NMES and NMES+PRO. Conversely, carbohydrate intake, physical activity, sedentary time, sleep duration, and sleep efficiency were not altered across the two interventions.

### 6.7 Participant perceptions of the intervention and adverse events

Quantitative data from the multiple‐choice component of the bespoke end‐of‐intervention questionnaire are reported in Table S4. The majority of the participants believed that the intervention had a positive impact on their life and that the virtual supervision positively contributed to their adherence. Comments from the open‐ended questions of the end‐of‐intervention questionnaire were varied, yet a prominent feature related to the poor reliability of the device. These were related to the stimulation device regularly cutting out during the training sessions, which occurred for nine of the participants, hampering the fluidity of the session. A handful of participants reported changes in spasms in the initial stages of training but these subsided and the participants were happy to continue training. One participant developed skin irritation from the electrode pads that persisted and led to drop out. Another participant experienced a suspected episode of Autonomic Dysreflexia after the first training session which led to removal from the study and reporting to the institutional ethics committee. No other adverse effects of training were observed (e.g., no electrical burns and no additional neuropathic pain).

## 7 DISCUSSION

Our study demonstrated that home‐based NMES‐RT is a feasible and efficacious method for increasing thigh and leg lean mass in persons with SCI, and that the increase in thigh lean mass is further enhanced by protein supplementation. Additionally, indices of insulin sensitivity were improved after 12 weeks of both NMES and NMES+PRO, although no additional benefit of protein supplementation was observed. Together, the findings of this pilot RCT indicate that NMES+PRO has potential as an accessible home‐based intervention to improve body composition and cardiometabolic outcomes in persons with SCI, warranting further investigation through a full‐scale RCT.

A substantial reduction in lower‐limb lean mass is one of the hallmarks of SCI (Biering‐Sorensen et al., 2009). The atrophied and disused muscle is further characterized by increased inflammation and a conversion from type IIa to type IIx fibers (Yarar‐Fisher et al., 2013). Nonetheless, our recent systematic review into methods to increase lean mass in persons with SCI highlighted that paralyzed muscle maintains the capacity for hypertrophy, and that NMES‐RT appears to be among the most effective strategies to promote muscle growth (Fenton et al., 2003). Indeed, thigh lean mass increased by ~3%–8% and leg lean mass by ~3%–5% after the intervention. These improvements are smaller than those reported in other NMES‐RT studies that used DXA to estimate lean mass. Ryan et al. (2013) found a ~ 10% increase in thigh lean mass following 16 weeks, twice per week NMES‐RT, while Legg Ditterline et al. (2020) reported a ~ 9% increase in leg lean mass. The longer duration of these interventions (16 vs. 12 weeks) may partly explain this discrepancy as Ryan et al. (2013) used a similar portable NMES device and stimulation parameters compared with the current study (e.g., 250 vs. 300 μs contraction duration). The training frequency may be an additional explanation, as previous studies investigated twice weekly training (Gorgey et al., 2017; Legg Ditterline et al., 2020; Mahoney et al., 2005; Ryan et al., 2013) compared with thrice weekly in the current study. (Mahoney et al., 2007) reported a higher level of neuromuscular fatigue following low‐frequency NMES compared with able‐bodied control individuals, suggesting that sub‐optimal recovery in between training sessions may have hampered the NMES‐RT‐induced hypertrophy. Finally, the NMES device used remains a potential explanation for the slightly reduced hypertrophy compared with other studies. Here we used the portable Compex Fit 3.0 to conduct NMES‐RT. While portable NMES devices can evoke a similar peak torque compared to laboratory‐based, clinical NMES devices (Lyons et al., 2005), their lower maximum amplitude may limit the ability of the paralyzed leg to reach full knee extension (Gorgey et al., 2017). For example, in the current study, four participants (two of each group) did not reach full knee extension at the start of the intervention and did not use ankle weights throughout the intervention. A discrepancy in efficacy between both modes of delivery has been noted previously by Gorgey et al. (2017), who found a ~ 10% increase in thigh skeletal muscle cross‐sectional area compared to >35% reported following laboratory‐based NMES‐RT (Fenton et al., 2003). Thus, home‐based NMES‐RT using a portable device increases leg lean mass, albeit of a smaller magnitude compared with laboratory‐based clinical devices. Considering the transportation and facilities barriers that people with SCI face (Martin Ginis et al., 2014), this is a trade‐off worthy of consideration for many individuals and health care providers.

Once‐daily protein supplementation potentiated the positive effects of NMES‐RT on thigh lean mass. Furthermore, participants in NMES+PRO were able to lift heavier ankle weights at the end of the intervention compared with NMES. No other studies have directly investigated the additive effect of protein supplementation in combination with resistance training on lean mass in this population. However, Kressler et al. (2014) found enhanced improvements in anaerobic and aerobic capacity when protein supplementation was consumed immediately before and after circuit training sessions as compared to when protein supplementation was delayed until the day after. Conversely, Yarar‐Fisher et al. (2018) found no effect of increased protein intake alone on leg lean mass and skeletal muscle composition. The additive effect of protein supplementation found in the current study is in line with studies investigating non‐injured adults (Morton et al., 2018; Tieland et al., 2012) and older adults (Finger et al., 2015; Liao et al., 2019). While pre‐clinical studies indicate that SCI may negatively affect muscle protein synthesis (Navegantes et al., 2004), the current data thus suggest that additional protein intake also enhances resistance training‐induced muscle protein synthesis after SCI in humans. Whether the magnitude of its effect is reduced in this population, as appears to be the case with aging and for those with a limited training history (Morton et al., 2018), cannot be discerned from these data and requires further study. Nonetheless, protein supplementation may be a low‐cost and easy‐to‐implement addition to an NMES‐RT intervention.

Skeletal muscle is the largest reservoir for glucose disposal in the body (DeFronzo & Tripathy, 2009). Therefore, increasing lean mass as well as its oxidative capacity through NMES‐RT (Yarar‐Fisher et al., 2018) was anticipated to translate to improvements in glucose tolerance and related cardiometabolic health outcomes. In the current study, both NMES and NMES‐RT led to improved insulin sensitivity in the fasted state, as indicated by a lower circulating insulin concentration for the same or lower glucose concentration and a lower HOMA‐IR, which likely reflects improved hepatic insulin sensitivity (DeFronzo & Tripathy, 2009). This effect may be related to the reduction in waist circumference in both groups, as visceral adiposity is strongly associated with glucose intolerance (Goodpaster et al., 1997). However, prandial glucose tolerance, which incorporates a larger component of skeletal muscle insulin sensitivity besides β‐cell function, adipose tissue and hepatic insulin sensitivity, and clearance (Norton et al., 2022), did not improve following either intervention. The lack of improvement is in contrast with some (Mahoney et al., 2005; Yarar‐Fisher et al., 2013) but not all (Gorgey et al., 2017; Ryan et al., 2013) other NMES‐RT studies. Mahoney et al. (2005) found a strong trend for improved prandial glucose tolerance, while Yarar‐Fisher et al. (2013) showed an increased expression of insulin signaling markers in skeletal muscle following a similar NMES protocol. It should be noted that Mahoney et al. (2005) studied only four participants; thus, the evidence for the benefits of NMES‐RT for prandial glucose tolerance remains limited. A test that more specifically targets skeletal muscle insulin sensitivity, such as a euglycemic‐insulin clamp, may provide more insight into the insulin‐sensitizing effect of NMES‐RT.

Finally, fasting LDL‐cholesterol and glucose concentration were reduced in NMES only. This is somewhat surprising given the larger increase in thigh lean mass, a trend for a larger reduction in waist circumference and body weight, as well as a higher concentration of total cholesterol, triglycerides, and LDL‐cholesterol at baseline in NMES+PRO. Considering these measures together, the lack of further improvement in cardiometabolic health following NMES+PRO compared to NMES suggests that although skeletal muscle plays a crucial role in these outcomes (DiMenna & Arad, 2018), a small difference in lean mass change as observed between both conditions is insufficient to induce additional benefits.

Adherence to the intervention was excellent (>96% for all intervention components), indicating that NMES‐RT and protein supplementation are feasible in a cohort with a wide range of levels of injury (T12–C5), and supporting the progression of this pilot study into a full‐scale RCT. Exit interviews showed that the majority believed the intervention had a positive impact on their life and that the virtual supervision aided their adherence. Considering the mixed adherence rates in laboratory‐based studies in persons with SCI (e.g., (Bakkum et al., 2015)), home‐based interventions with remote supervision may have the potential to increase the uptake of healthy behaviors in this population. However, it should be noted that the portable NMES devices frequently cut out, which interrupted the training sessions; something that nine participants in their exit interview also noted. Furthermore, despite a medical review before enrolment, one episode of autonomic dysreflexia occurred during the home‐based sessions. While the episode was mild and resolved when the stimulation was ceased, this demonstrates that safety should remain a consideration with this activity. Therefore, remote supervision and/or a caregiver to be present, or initial practice sessions in a laboratory environment are aspects that may need to be part of future investigations of this intervention.

As with any research, this study has limitations and has exposed considerations for future work. The current study did not include a non‐exercise control group, a caveat that should be kept in mind when appraising the effects of NMES per se. In addition, there were differences in some of the baseline characteristics and cardiometabolic health parameters between both groups, which is reflective of the heterogonous nature of SCI and the small sample size of this pilot study. We have minimized its impact on the outcomes by controlling for the baseline value of the parameter of interest and conducting a sensitivity analysis of the primary outcome using an established multiple imputation method in SCI populations (Yi et al., 2006). Nonetheless, it is worth noting that Gorgey et al. (2023) found that a higher body weight at baseline, which was found in the NNMES+PRO group, appears beneficial for hypertrophic adaptations to NMES‐RT. Future studies may also consider exploring different portable devices that are more dependable for home use. Finally, when investigating individuals with sufficient upper‐limb function, upper‐body resistance exercise could be included to enhance the impact of the intervention on whole body parameters.

## 8 CONCLUSIONS

In conclusion, 12 weeks of home‐based NMES‐RT increased thigh and leg lean mass, an effect that was potentiated by protein supplementation. Furthermore, both NMES and NMES+PRO improved indices of glucose tolerance, with no added benefit of protein supplementation. These findings indicate that home‐based NMES+PRO is a feasible and promising intervention to improve body composition and cardiometabolic health outcomes in persons with SCI that warrants further investigation through a full‐scale RCT.

## AUTHOR CONTRIBUTIONS

Sven Hoekstra: Conceived and designed research, performed experiments, interpreted results of experiments, drafted manuscript, edited and revised manuscript, approved final version of manuscript. James A. King: Conceived and designed research, interpreted results of experiments, drafted manuscript, edited and revised manuscript, approved final version of manuscript. Jordan Fenton: Conceived and designed research, performed experiments, analyzed data, interpreted results of experiments, approved final version of manuscript. Natasha Kirk: performed experiments, analyzed data, interpreted results of experiments, prepared figures, edited and revised manuscript, approved final version of manuscript. Scott A. Willis: analyzed data, interpreted results of experiments, prepared figures, edited and revised manuscript, approved final version of manuscript. Stuart M. Phillips: Conceived and designed research, interpreted results of experiments, edited and revised manuscript, approved final version of manuscript. Nick Webborn: medical oversight, approved final version of manuscript. Keith Tolfrey: analyzed data, interpreted results of experiments, approved final version of manuscript. Johan De Vogel‐Van Den Bosch: Conceived and designed research, interpreted results of experiments, edited and revised manuscript, approved final version of manuscript. Vicky L. Goosey‐Tolfrey: Conceived and designed research, interpreted results of experiments, drafted manuscript, edited and revised manuscript, approved final version of manuscript.

## FUNDING INFORMATION

The protein supplements and financial support for the biochemical analyses were provided by Danone Research & Innovation.

## ETHICS STATEMENT

The study was approved by Loughborough University Ethics Review Sub‐Committee (Project ID:3176).

## DISCLOSURES

J dV‐VdB is employed by Danone Research & Innovation.

## Supporting information


Data S1:

## Data Availability

Data are available upon reasonable request.
