# Supplementary material for: The effect of home‐based neuromuscular electrical stimulation‐resistance training and protein supplementation on lean mass in persons with spinal cord injury: A pilot study
Source: Physiol Rep. 2024 Oct 2;12(19):e70073. doi: 10.14814/phy2.70073 (PMC11446856; doi:10.14814/phy2.70073)
Supplement: Supplementary file 1 — Data S1: [file PHY2-12-e70073-s001.docx]

**Supplementary Table 1.** Nutritional composition of the protein supplementation ingested^1^ during the 12-week intervention for the (NMES-RT) + PRO group in spinal cord injured men and women.

| **Component** | **Protein** | |
| --- | --- | --- |
| Energy, kJ | 628.0 |  |
| Energy, kcal | 150.0 |  |
| Protein, g | 20.7 |  |
| EAAs, g | 10.6 |  |
| Leucine, g | 2.8 |  |
| Carbohydrate, g | 9.4 |  |
| Fat, g | 3.0 |  |
| Fiber, g  Cholecaliciferol, µg | 1.3  20.0 |  |
|  |  | |

**Note.** Neuromuscular electrical stimulation, resistance training (NMES-RT); ^1^ Dried beverage contents were dissolved in 250 mL water. EAA, essential amino acid (Leu, Ile, Val, Phe, Met, His, Trp, Thr, and Lys) in vanilla of strawberry flavors. The protein drink also contained other micro nutrients: sodium(150mg), potassium (279mg), chloride (70mg), calcium (500mg), phosphorus (250mg), magnesium (37mg), iron (2.4mg), zinc (2.2mg), copper (270µg), manganese (0.5mg), fluoride (0.15mg), molybdenum (15µg), selenium (15µg), chromium (7.5µm), iodine (20µg), vitamin A (152µg), vitamin E (7.5mg), phylloquinone (12µg), thiamine (0.23mg), riboflavin (0.25mg), nicotinic acid (8.8mg), pantothenic acid (0.81mg), pyridoxine (0.76mg), folic acid (203µg), cyanocobalamin (3µg), biotin (6.1µg), ascorbic acid (32mg), provitamin A carotenoids (0.3mg), and choline (56mg).

**Supplementary Table 2.** Habitual physical activity, sedentary time, sleep and dietary characteristics of the NMES and NMES+PRO groups.

| **Variable** | **NMES** | **NMES+PRO** |
| --- | --- | --- |
| **Physical activity, sedentary time and sleep** | | |
| Light PA (min∙d^-1^) | 257 ± 94 | 241 ± 120 |
| Moderate PA (min∙d^-1^) | 64 ± 43 | 44 ± 49 |
| Vigorous PA (min∙d^-1^) | 1 (0,5) | 10 (0,23) |
| Sedentary time (min∙d^-1^) | 636 ± 144 | 659 ± 161 |
| Sleep duration (min∙d^-1^) | 410 ± 55 | 438 ± 35 |
| Sleep efficiency | 0.88 ± 0.08 | 0.87 ± 0.05 |
| **Dietary intake** |  |  |
| Energy intake (kcal∙d^-1^) | 1701 ± 445 | 1582 ± 1036 |
| Absolute protein intake (g∙d^-1^)* | 85 (60, 97) | 57 (37, 100) |
| Relative protein intake (g∙kg^-1^∙d^-1^) | 1.2 ± 0.3 | 0.9 ± 0.7 |
| Absolute fat intake (g∙d^-1^) | 75 (62, 86) | 51 (38, 106) |
| Relative fat intake (g∙kg^-1^∙d^-1^) | 1.0 ± 0.3 | 0.9 ± 0.7 |
| Absolute CHO intake (g∙d^-1^) | 169 ± 68 | 161 ± 93 |
| Relative CHO intake (g∙kg^-1^∙d^-1^) | 2.4 ± 1.0 | 1.9 ± 1.2 |

Data are presented as mean SD, or as median (interquartile range) where data were non-normally distributed. For the NMES and NMES+PRO groups respectively, *n* = 7 and *n* = 6 for dietary intake data and *n* = 5 and *n* = 7 for physical activity, sedentary time and sleep data. CHO, carbohydrate; NMES, neuromuscular electrical stimulation; NMES+PRO, neuromuscular electrical stimulation + protein supplementation; PA, physical activity.

* Note. The participants in NMES+PRO group did not report the protein supplementation in their food diary.

**Supplementary Table 3.** Pre- to post- intervention (within-group) changes and the between group differences (NMES+PRO minus NMES) in physical activity, sedentary time, sleep and dietary intake after the 12-week intervention period.

| **Variable** | **Mean change from baseline (post minus pre)** | | | | **Between-group difference (NMES+PRO minus NMES) (95% CI)** | ***P*-value** | **Effect size (*d*)** |
| --- | --- | --- | --- | --- | --- | --- | --- |
|  | **NMES** | | **NMES+PRO** | |  |  |  |
|  | ***n*** | **Mean change (95% CI)** | ***n*** | **Mean change (95% CI)** |  |  |  |
| **Physical activity, sedentary time and sleep** | | | | | | | |
| Light PA (min∙d^-1^) | 5 | -2 (-54, 51) | 7 | -31 (-75, 14) | -29 (-98, 40) | 0.410 | 0.48 |
| Moderate PA (min∙d^-1^) | 5 | -16 (-33, 0) | 7 | -11 (-25, 3) | 5 (-16, 27) | 0.612 | 0.30 |
| Vigorous PA (min∙d^-1^) | 5 | -4 (-10, 2) | 7 | 1 (-4, 6) | 5 (-3, 14) | 0.221 | 0.76 |
| Sedentary time (min∙d^-1^) | 5 | 33 (-27, 93) | 7 | 34 (-16, 85) | 1 (-77, 80) | 0.976 | 0.02 |
| Sleep duration (min∙d^-1^) | 5 | -14 (-66, 39) | 7 | -33 (-77, 11) | -19 (-88, 50) | 0.589 | 0.32 |
| Sleep efficiency | 5 | 0.01 (-0.07, 0.01) | 7 | -0.06 (-0.12, 0.01) | -0.07 (-0.17, 0.04) | 0.236 | 0.69 |
| **Dietary intake** | | | | | | | |
| Energy intake (kcal∙d^-1^) | 7 | -265 (-528, -2) | 6 | -201 (-464, 62) | 64 (-309, 427) | 0.737 | 0.18 |
| Absolute protein intake  (g∙d^-1^) | 7 | -19 (-36, -1) | 6 | -16 (-33, 1) | 3 (-22, 28) | 0.818 | 0.12 |
| Relative protein intake  (g∙kg^-1^∙d^-1^) | 7 | -0.2 (-0.4, 0.1) | 6 | -0.3 (-0.5, 0.0) | -0.1 (-0.4, 0.2) | 0.545 | 0.33 |
| Absolute fat intake (g∙d^-1^) | 7 | -16 (-30, -2) | 6 | -19 (-33, -5) | -3 (-23, 17) | 0.787 | 0.15 |
| Relative fat intake  (g∙kg^-1^∙d^-1^) | 7 | -0.2 (-0.3, 0.0) | 6 | -0.3 (-0.5, -0.1) | -0.1 (-0.3, 0.1) | 0.340 | 0.51 |
| Absolute CHO intake (g∙d^-1^) | 7 | -8 (-31, 15) | 6 | -3 (-26, 20) | 5 (-27, 37) | 0.760 | 0.16 |
| Relative CHO intake  (g∙kg^-1^∙d^-1^) | 7 | -0.1 (-0.3, 0.2) | 6 | -0.1 (-0.4, 0.2) | 0.0 (-0.4, 0.4) | 0.830 | 0.12 |

Data were analyzed using generalized linear models with a normal distribution and identity link function and are presented as mean change from baseline adjusted for pre-intervention values with 95% CI. CHO, carbohydrate; CI, confidence interval; NMES, neuromuscular electrical stimulation; NMES+PRO, neuromuscular electrical stimulation + protein supplementation; PA, physical activity.

**Supplementary Table 4.** Participants perceptions of the intervention (data collected at the follow-up laboratory trial). Q9-Q11 for NMES+PRO group only.

|  | **Strongly disagree** | **Disagree** | **Neither agree nor disagree** | **Agree** | **Strongly agree** |
| --- | --- | --- | --- | --- | --- |
|  | **(no. of participants selecting this answer; n = 15)** | | | | |
| Q1) I enjoyed this training program. | 0 | 0 | 1 | 4 | 10 |
| Q2) I feel like the effects of this training had a positive effect on other elements of my day-to-day life. | 0 | 2 | 4 | 6 | 3 |
| Q3) I feel like having a virtual supervisor made me more likely to stick to the training program. | 1 | 1 | 3 | 3 | 7 |
| Q4) If I had all of the equipment, I would continue to do this training. | 0 | 2 | 3 | 3 | 7 |
| Q5) I feel like the effects of this training had a negative effect on other elements of my day-to-day life. | 8 | 5 | 2 | 0 | 0 |
| Q6) I feel like this training program took up too much of my time. | 7 | 8 | 0 | 0 | 0 |
| Q7) I feel like I’ve had a very tiring workout after doing this training. | 7 | 4 | 3 | 0 | 1 |
| Q8) The device was easy to use. | 1 | 2 | 1 | 10 | 1 |
|  | **(no. of participants selecting this answer; n = 8)** | | | | |
| Q9) I found the protein supplement tasty. | 1 | 2 | 1 | 3 | 0 |
| Q10) I found it difficult having to take the protein supplement every day. | 2 | 4 | 1 | 1 | 0 |
| Q11) The protein supplement made me feel sick. | 5 | 3 | 0 | 0 | 0 |
